# Depletion of muscularis macrophages ameliorates inflammation-driven dysmotility in murine colitis model

**DOI:** 10.1038/s41598-023-50059-7

**Published:** 2023-12-17

**Authors:** Szilamér Ferenczi, Fruzsina Mogor, Peter Takacs, Tamas Kovacs, Viktoria E. Toth, Zoltán V. Varga, Krisztina Kovács, Zoltan Lohinai, Koppány Csaba Vass, Nandor Nagy, David Dora

**Affiliations:** 1 Institute of Experimental Medicine, Laboratory of Molecular Neuroendocrinology, Budapest, Hungary; 2 Institute of Genetics and Biotechnology, Department of Microbiology and Applied Biotechnology, Hungarian University of Agriculture and Life Sciences, Gödöllő, Hungary; 3 Department of Anatomy, Histology and Embryology, Semmelweis University, Tuzolto St. 58, Budapest, 1094 Hungary; 4 Department of Pharmacology and Pharmacotherapy, Semmelweis University, Budapest, Hungary; 5 MTA-SE Momentum Cardio-Oncology and Cardioimmunology Research Group, Budapest, Hungary; 6 Translational Medicine Institute, Semmelweis University, Budapest, Hungary; 7 Department of Laboratory Medicine, Semmelweis University, Budapest, Hungary

**Keywords:** Immunology, Physiology, Gastroenterology

## Abstract

Previously, the presence of a blood-myenteric plexus barrier and its disruption was reported in experimentally induced colitis via a macrophage-dependent process. The aim of this study is to reveal how myenteric barrier disruption and subsequent neuronal injury affects gut motility in vivo in a murine colitis model. We induced colitis with dextran sulfate sodium (DSS), with the co-administration of liposome-encapsulated clodronate (l-clodronate) to simultaneously deplete blood monocytes contributing to macrophage infiltration in the inflamed muscularis of experimental mice. DSS-treated animals receiving concurrent l-clodronate injection showed significantly decreased blood monocyte numbers and colon muscularis macrophage (MM) density compared to DSS-treated control (DSS-vehicle). DSS-clodronate-treated mice exhibited significantly slower whole gut transit time than DSS-vehicle-treated animals and comparable to that of controls. Experiments with oral gavage-fed Evans-blue dye showed similar whole gut transit times in DSS-clodronate-treated mice as in control animals. Furthermore, qPCR-analysis and immunofluorescence on colon muscularis samples revealed that factors associated with neuroinflammation and neurodegeneration, including Bax1, Hdac4, IL-18, Casp8 and Hif1a are overexpressed after DSS-treatment, but not in the case of concurrent l-clodronate administration. Our findings highlight that MM-infiltration in the muscularis layer is responsible for colitis-associated dysmotility and enteric neuronal dysfunction along with the release of mediators associated with neurodegeneration in a murine experimental model.

## Introduction

The enteric nervous system (ENS) as part of the autonomic nervous system comprises a vast network of neural and glial cells that performs a wide array of independent functions^1,2^, like regulating gastrointestinal (GI) motility and participating in a crosstalk with the microbiota and the immune system of the gut^3,4^. In the recent decade, our knowledge on the cellular-biochemical crosstalk between the microbiome, muscularis macrophages (MMs) and the ENS has been expanded exceptionally^5,6^. Inflammatory bowel diseases, such as ulcerative colitis and Crohn's disease, place a heavy burden on health care systems, with approximately 30 million patients worldwide^7^. These patients often complain about symptoms associated with motility dysfunction (diarrhea, constipation) between acute episodes^8,9^.

During gangliogenesis migrating enteric neural crest cells secrete different extracellular matrix (ECM) proteins in the embryonic gut, like collagens, tenascin and agrin^10,11^ that persist also in adult life separating the neural microenvironment in the muscularis externa^12^. Other key players in gut neuroimmunology, intestinal macrophages, colonize every layer of the GI tract, including the muscularis externa^3,13^. MMs represent a homogeneous population of MHCII^high^CD11c^low^CD103^−^CD11b^+^ cells localized in the circular and longitudinal muscle layer and associated to the myenteric plexus, expressing high levels of CX3CR1^13^. It was also shown that MMs are highly dependent on Colony stimulating factor 1 receptor (CSF1R), but not mucosal macrophages, based on antibody-based depletion methods^3^. A recent study implicated that a population of MMs, closely associated to the ENS forms a radioresistant, self-renewal cell population, similar to CNS microglia. The selective inhibition of these cells reduces GI motility by hindering ENS function^5,14^. Moreover, a complex, new reciprocal cell–cell communication via TGFβ was described lately, that is responsible for ENS homeostasis and indicates that similarly to the brain, is shaped and maintained by a dedicated population of resident macrophages^15^.

Recently, we showed that enteric ganglia and the microenvironment of the ENS is protected by an impermeable barrier built from glial endfeet and a basement membrane rich in ECM proteins, including heparan-sulfate proteoglycan agrin and collagen type 4^11,16^. DSS-induced colitis disrupts this myenteric plexus barrier increasing its permeability in a macrophage-dependent manner^16^. DSS (dextran sulfate sodium) is a tight junction poison that induces colonic inflammation and bloody diarrhea by disrupting the integrity of the epithelial barrier^17^. This can be prevented with the concurrent administration of liposome-encapsulated (l-)clodronate and the consequent depletion of MMs from the muscularis^16,18^. Enteric neuroinflammation and neuronal injury are well-known phenomena in IBD and experimental colitis models^19–22^ that can be a direct consequence of myenteric plexus barrier disruption^16^. It was also implicated that apart from inflammation, simultaneous oxidative stress^23^ and hypoxia^24^ are also responsible for the pathological and physiological consequences of IBD, including loss of enteric neurons and dysmotility.

Previous studies already examined the relation between MMs and motility in pathological conditions, such as surgical or septic ileus in a iNOS-KO model^25^, or by inhibiting monocyte chemoattractant protein 1 (MCP1) in a trinitrobenzenesulfonic acid (TNBS)-induced colitis model in rats^26^. We have already gained knowledge about the fact, that acute inflammation could inflict long-term changes in the electrophysiological properties of enteric neurons in the myenteric-^27^ and submucosal plexi^28^, however, the involvement of MMs and the myenteric plexus barrier has not yet been elucidated in this process.

To explore whether the retainment of the barrier during intestinal inflammation can prevent colitis-induced dysmotility, we performed motility measurements on control-, DSS-treated and DSS + l-clodronate treated mice. To reveal whether experimental macrophage-depletion can alleviate neuronal injury, we performed qPCR and Immunofluorescence (IF) on colon muscularis samples to assess the expression of neurogenic factors directly involved in their injury and inflammation, based on bulk RNAseq results. Our study aimed to examine the link between the degradation of the myenteric plexus barrier, and neuroinflammation associated dysmotility the first time in an experimental animal model.

## Results

### Liposomal clodronate treatment ameliorates DSS-induced murine colitis and depletes infiltrating macrophages from the muscularis externa

**Figure 1:**
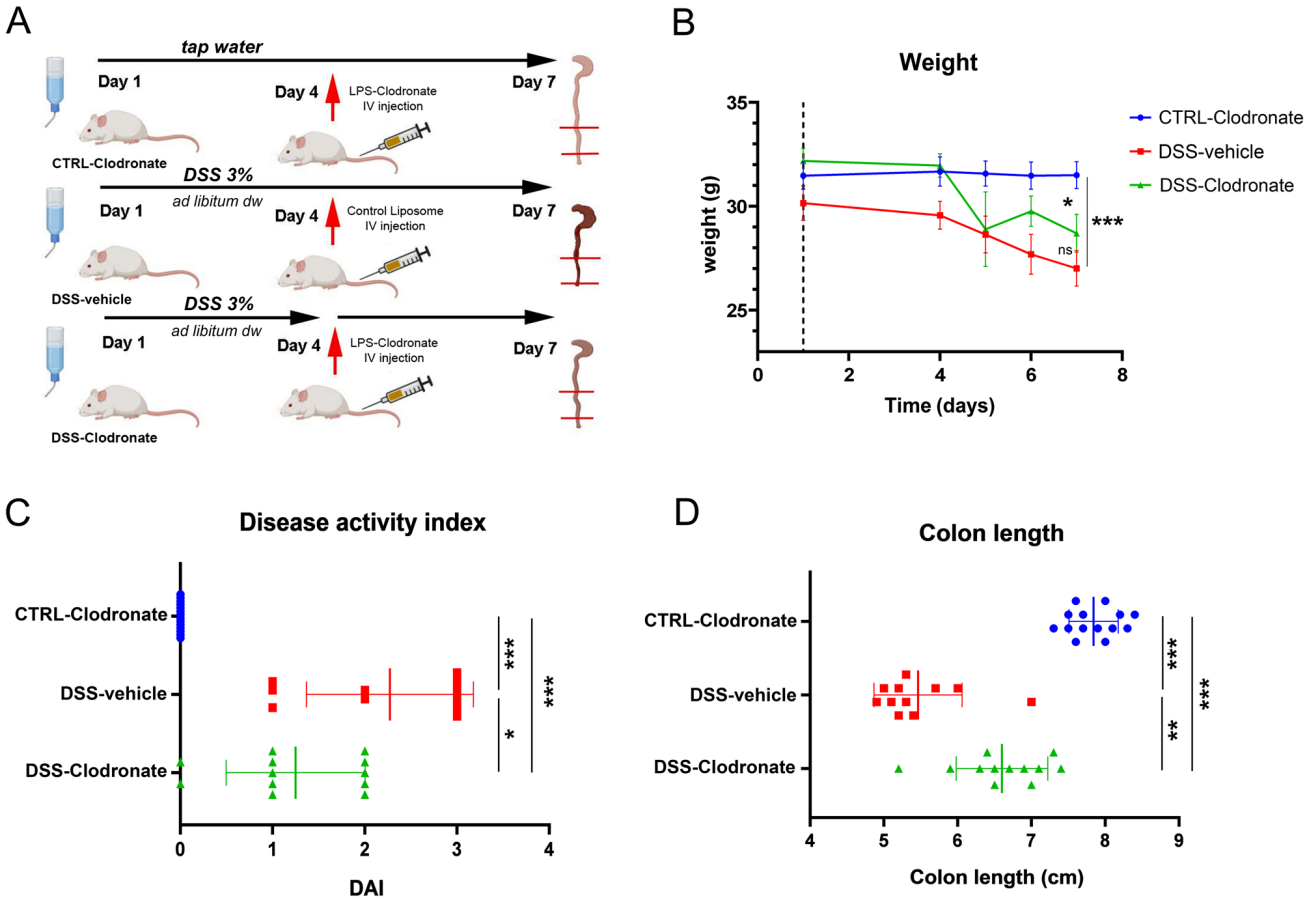
Experimental design and colitis phenotype. Illustrated flowchart demonstrated study design (**A**), where experimental groups of CTRL-clodronate, DSS-vehicle and DSS-clodronate-treated mice are shown. Weight (grams) of animals monitored during the experiment is shown in (**B**). Weight of DSS-vehicle-treated mice decreased significantly, by 22.9% (p < 0.001) as expected, but was not significantly different from those who received l-clodronate during DSS-treatment (p = 0.076). A moderate, but significant weight-loss (17.5%) occurred in DSS-clodronate-treated mice compared to control littermates (p = 0.012). Disease activity index (DAI) was significantly higher in the DSS-vehicle group compared to the control (p < 0.001) and DSS-clodronate (p = 0.014) treated experimental groups. The latter showed a significant difference compared to the control group (p < 0.001) as well (**C**). Colon of DSS-vehicle-treated animals were significantly shortened compared to control (p < 0.001) and DSS-clodronate treated animals (p = 0.001), but colon length of the latter was also significantly shorter compared to that of control mice (p < 0.001) (**D**). Metric data are shown as mean and corresponding standard deviation (SD). Statistical significance *p < 0.05; **p < 0.01, ***p < 0.001.

In our experimental model, 100-day-old male FVB/Ant mice were culled into three experimental groups: from day 1, the control group received tap water, whereas the two experimental groups received 3% DSS in their drinking water. On day 4, all animals received injections into their tail vein. The control group (CTRL-clodronate) and the DSS-clodronate group received l-clodronate, while the DSS-vehicle group received control liposomes devoid of clodronate. All animals continued the original diet until day 7. After clinical and motility assessments, mice were sacrificed and their colon processed for histology (Fig. 1A). l-clodronate shows no systemic toxic effect, but induces apoptosis in all mononuclear cells capable of phagocytosis^18^. Since liposome size prevents its transport through continuous endothelium, depletion only takes place in the bloodstream, the bone marrow, the liver and the spleen having discontinuous endothelial linings^29^. Experimental design is shown in Fig. 1A.

Weight of DSS-vehicle-treated animals decreased significantly compared to control animals, but showed no significant difference compared to the DSS-clodronate group at the end of the experiment (Fig. 1B). Assessment of disease activity index (DAI)—describing the extent of hematochezia and general health status—showed that DSS-clodronate-treated mice exhibited a milder disease phenotype, but still symptomatic contrary to control animals (Fig. 1C). Colon length is used as another reliable marker of colitis severity in murine DSS models^16,17^, where reactive shortening of distal bowel segments may indicate inflammation activity. Similar to DAI, evaluation of colon length indicated a severe disease phenotype in DSS-vehicle treated mice, and an intermediate disease phenotype in DSS-clodronate treated animals (Fig. 1D).

To confirm that non-inflamed colon of l-clodronate-treated mice exhibit no macrophage-depletion compared to control mice, we performed immunofluorescence (IF) on distal colon, and immunohistochemistry (IHC) on liver and spleen tissues of animals. As expected, l-clodronate treated animals show no signs of macrophage depletion in the gut compared to control animals (SFig 1A,A′), however, a significantly decreased number of macrophages are present in the spleen (SFig 1B,B′) and liver (SFig 1C,C′) of l-clodronate treated mice.

### L-clodronate treatment depletes Ly6C+ monocytes, but not granulocytes from the blood

**Figure 2:**
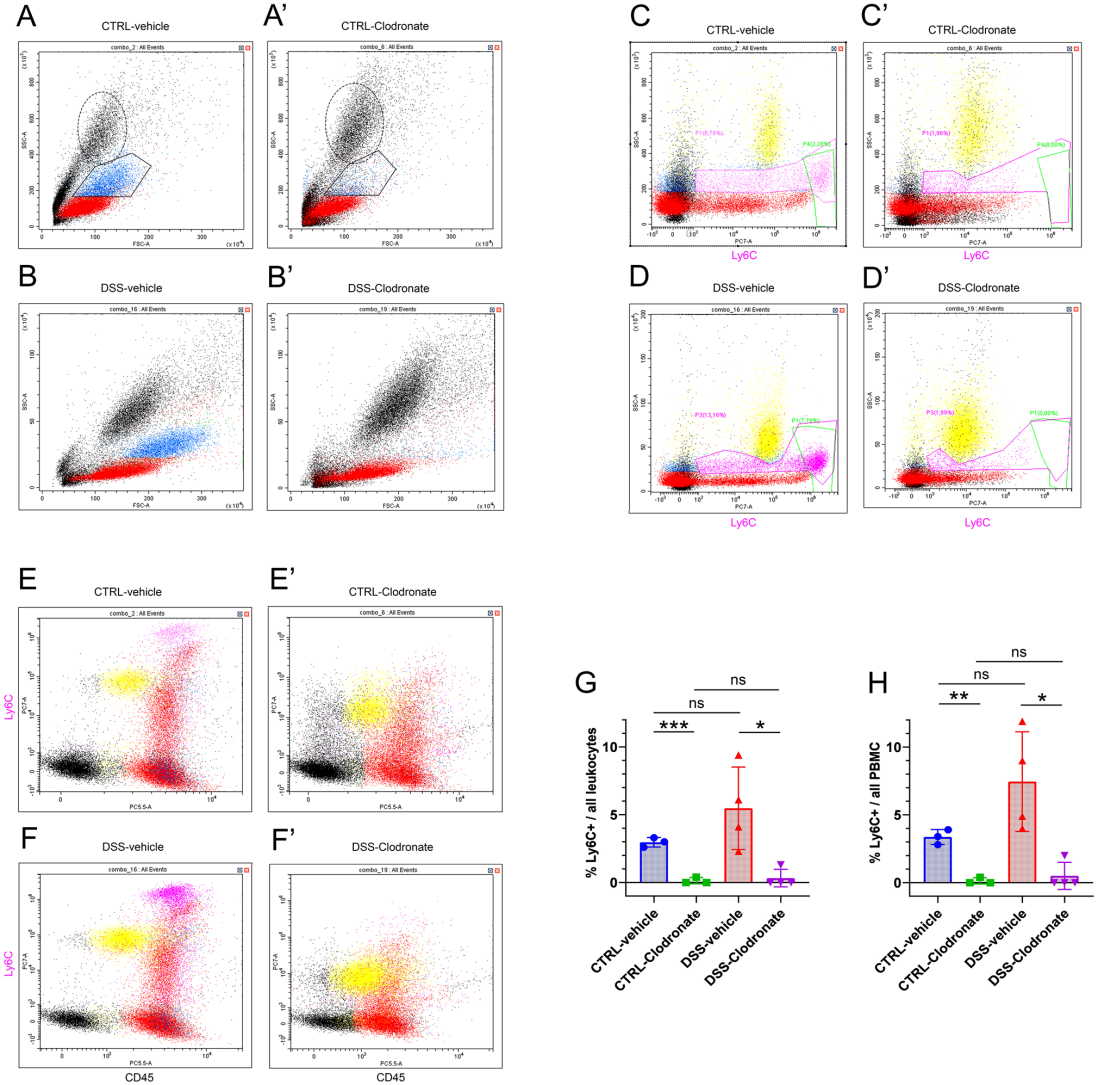
Flow cytometry analysis of experimental mouse blood samples. Scatter plots show all events displaying cells according to SSC (axis y) and FSC (axis x). PBMCs are colored as lymphocytes (red) and monocytes (blue). Supposed granulocyte population is labeled with oval dashed curve, showing no alteration due to treatment (**A**–**B**′). Depletion of monocytes after l-clodronate treatment is shown with rectangles (**A**,**A**′). SSC-Ly6C plots display cells according to their expression of the monocyte marker (x axis) showing a “high” (green rectangle, > 10^6^), a “low” (10^3^–10^6^) and a “negative” (< 10^3^) population. Magenta rectangle encircles Ly6C-positive cells including all “low” and “high” cells (**C**–**D**′). Interestingly, a fraction of lymphocytes and all granulocytes express a low level of Ly6C (**C**–**D**′). Panels (**E**–**F**′) exhibit cell distribution according to their CD45 (x axis) and Ly6C (y axis) expression. Double negative events on the bottom left are identified as cell debris and thrombocytic fragments. Bar charts on panels (**G**) and (**H**) show the comparison of Ly6C^high^ monocytes compared to all leukocytes (PBMCs + granulocytes) identified among events and to PBMCs identified. A significantly lower percentage of Ly6C+ monocytes were identified in both control and DSS-treated experimental conditions when calculating for all leukocytes (p < 0.001 and p = 0.041) and for all PBMCs (p = 0.004 and p = 0.028). However, no significant change, only a trend was detected when comparing control- and DSS-vehicle mice (p = 0.197 and p = 0.111, respectively), and l-clodronate treated animals from the control and DSS-groups showed no significant differences in the percentage of Ly6C^high^ cells (p = 0.523 and p = 0.614, respectively). (**A**–**B**′) Cells in blue: monocyte part of PBMC population based on SSC-FSC diagram; cells in red: lymphocyte part of PBMC population based on SSC-FSC diagram. Rectangle: monocytes, oval dashed line: granulocyte population based on SSC-FSC diagram. (**C**–**F**′) cells in blue: Ly6C− monocytes; cells in red: lymphocytes; cells in magenta: Ly6C+ monocytes (“high” or “low”); cells in yellow: granulocytes; cells in black: debris, thrombocytes. Metric data are shown as mean and corresponding standard deviation (SD). Statistical significance *p < 0.05; **p < 0.01, ***p < 0.001.

Previous findings have implicated that intravenous l-clodronate induces cell death in bone marrow and blood monocytes within 24 h, however these results were not confirmed independently with quantitative analysis^16,18,29^. Therefore we performed flow cytometry on the blood of control- and DSS-treated-mice, along with their l-clodronate-depleted counterparts. As previously described, on the 4th day of DSS-induction, n = 4 mice were treated with l-clodronate and n = 4 mice with vehicle (same procedure performed for 3–3 control mice). 24 h later, 1 ml of fresh blood was collected from the retroorbital venous plexus from every animal, then forwarded the same day to flow cytometry analysis. Side scatter (SSC) vs forward scatter (FSC) plots show the unambiguous effect of l-clodronate on the monocytic cell population (Fig. 2A,A′, cells in blue, rectangle) leaving the granulocyte population (Fig. 2A,A′, dashed circle) seemingly intact. A similar effect is visible when comparing DSS-vehicle and DSS-Clodronate-treated animals (Fig. 2B,B′). Lymphocytes, as expected, showed no significant numeral alteration after treatment (Fig. 2A–B′, cells in red). Apart from the SSC-FSC parameter combination, anti-Ly6C antibody was used to identify blood monocytes, which was reported to label 90% of this population in steady state^30^. In the blood, we identified a Ly6C^low^ population showing strong overlap with lymphocytes, and a Ly6C^high^ population made entirely of monocytes (Fig. 2C–D′, green rectangle). While the Ly6C^low^ population exhibits a significant drop in both groups of l-clodronate-administered animals, Ly6C^high^ monocytes are completely depleted from the blood (Fig. 2C–D′). Interestingly, the Ly6C-negative monocyte population (Fig. 2C–D′, cells in blue) shows a marked decrease in numbers too, but not lymphocytes or granulocytes (Fig. 2C–D′, cells in yellow).

Scatter plots in Fig. 2E–F′ show cellular distribution according to CD45 and Ly6C expression and the disappearance of the CD45^high^-Ly6C^high^ populations in l-clodronate-treated animals. The percentage of Ly6C^high^ monocytes among all leukocytes (Fig. 2G) and all peripheral blood mononuclear cells (PBMCs, Fig. 2H) decreases significantly both in control- (p < 0.001 and p = 0.004) and in DSS-treated conditions (p = 0.041 and p = 0.028). The fraction of Ly6C^high^ monocytes was not significantly elevated during inflammation, only a trend was observed both among all leukocytes and PBMCs (p = 0.197 and p = 0.111, Fig. 2G,H). The number of blood granulocytes did not show a substantial shift apart from a relative growth due to decreased number of cells in total, supporting the observation that l-clodronate treatment does not affect blood granulocytes (SFig 2).

### Administration of l-clodronate depletes MMs from the muscularis externa, but not from the mucosa, and disrupts the myenteric plexus barrier

**Figure 3:**
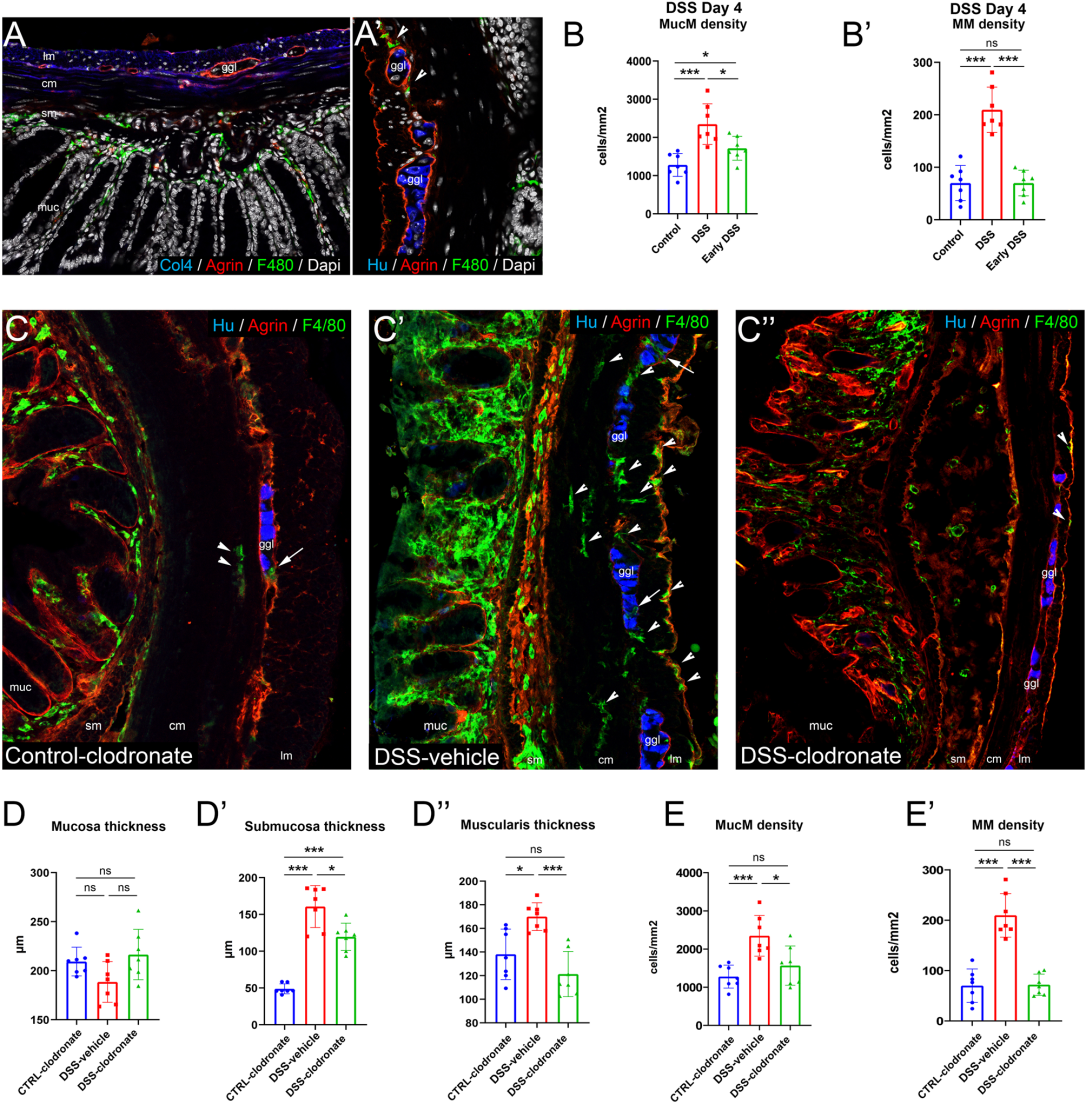
The effect of DSS and DSS + l-clodronate treatment on the mouse colon. IF stainings show colon sections of mice euthanized at the 4th day of DSS-treatment, where ECM molecule agrin delineate ganglia with intact myenteric plexus barriers. F4/80+ macrophages are already infiltrated the mucosa, but not the muscularis at this timepoint (**A**,**A**′). Arrows indicate scattered MMs in early DSS colon (**A**′). Cell counting shows that a significantly increased number of mucosal macrophages (MucM) are present in DSS- (8th day) compared to 4th day DSS- (p = 0.037) and control conditions (p < 0.001), likewise between control- and 4th day DSS conditions (p = 0.017) (**B**,**B**′). Panels (**C**–**C**″) show morphology of control (**C**), DSS-vehicle-treated (**C**′) and DSS-clodronate-treated (**C**″) colon stained with IF using antibodies Hu (enteric neurons), agrin and F4/80. Arrowheads indicate MMs, arrows indicate intraganglionic macrophages within the agrin-labeled myenteric plexus barriers. Bar charts show the results of morphometry and cell counting in the same experimental setting (**D**–**E**′). There was no significant difference in mucosa thickness in any comparison (**D**). Colon submucosa was significantly thicker in DSS- (p < 0.001) and DSS-clodronate treated mice (p < 0.001) compared to control littermates. For the same parameter, there was a modest, but significant difference between DSS- and DSS-clodronate treated animals (p = 0.026) (**D**′). The muscularis was significantly thicker in DSS-vehicle-treated animals compared to DSS-clodronate-treated (p < 0.001) and control mice (p = 0.011). There was no significant difference between the control and DSS-clodronate-treated groups. MucM density was significantly increased in DSS-vehicle-treated animals compared to DSS-clodronate-treated (p = 0.026) and control mice (p < 0.001). There was trend towards increased MucM density in the DSS-clodronate group vs the control group, but it did not reach statistical significance (**E**). MM density of DSS-vehicle-treated animals was significantly increased compared to both control (p < 0.001) and DSS-clodronate-treated animals (p < 0.001), but showed no significant difference between the control and the DSS-clodronate-treated groups (**E**′). Metric data are shown as mean and corresponding standard deviation (SD). Statistical significance *p < 0.05; **p < 0.01, ***p < 0.001. *ggl* enteric ganglion, *lm* longitudinal layer of muscularis externa, *cm* circular layer of muscularis externa, *muc* mucosa, *sm* submucosa.

To confirm that infiltration of immune cells occurs at later stages of the 7-day protocol, we sacrificed DSS-treated mice on the 4th day of treatment, the same time when l-clodronate is administered in other experiments. At this time, only the mucosa shows a modest increase in immune cell infiltration, there is no inflammatory infiltration detected in the muscularis (Fig. 3A,B). MMs are present in the muscularis layer, but in the same numbers as in the case of control specimens (Fig. 3A′,B′). This might mean that inflammation spreads in a centrifugal manner, and the delayed l-clodronate administration is more selective to deplete immune cells in the muscularis, thus, the experimental model is particularly useful to study the effect of MMs and infiltrating immune cells on the myenteric plexus barrier.

Histological analysis shows signs of modest inflammation, localized to the mucosal and submucosal layers in DSS + l-clodronate treated mice, in contrast with a severe inflammatory phenotype present in DSS-vehicle treated animals (Fig. 3C–C″). There are MMs only scattered in the muscularis layer of CTRL-clodronate (Fig. 3C, arrowheads) and DSS-clodronate (Fig. 3C″, arrowheads) mice, and a massive infiltration of MMs in DSS-vehicle treated animals (Fig. 3C′, arrowheads). The myenteric plexus barriers of enteric ganglia delineated with agrin expression are mostly degraded or semi-degraded in the inflamed colon (Fig. 3C′), whereas they are mostly intact in CTRL-clodronate (Fig. 3C) and DSS-clodronate animals (Fig. 3C″). At the end of the 7-day treatment, the colon of DSS-clodronate treated mice exhibited no significant change in mucosa thickness (Fig. 3D), modestly decreased submucosa thickness (Fig. 3D′) and significantly decreased muscularis thickness (Fig. 3D″) compared to DSS-vehicle treated animals. While the density of mucosal macrophages decreased only slightly- (Fig. 3E), the MM density decreased significantly in DSS-clodronate treated mice compared to their DSS-vehicle treated counterparts (Fig. 3E′).

### L-clodronate treatment reduces dysmotility in DSS-treated mice

**Figure 4:**
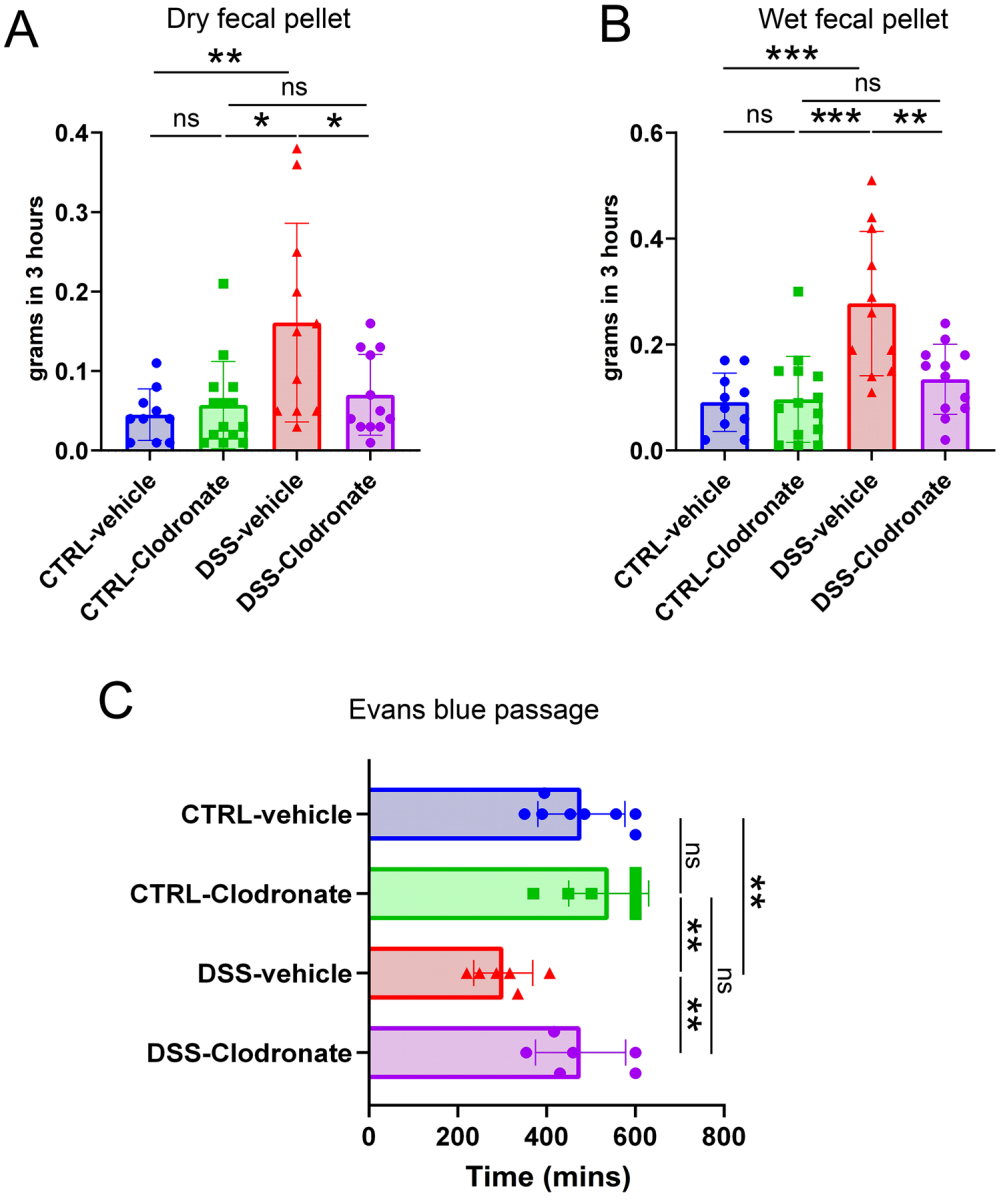
Results of motility measurements. Bar charts show outcome of motility studies, including the measurement of dry- (**A**) and wet (**B**) fecal pellet weight in Control-vehicle-treated, Control-clodronate-treated, DSS-vehicle-treated and DSS-clodronate-treated mice. The weight of dry fecal pellets collected in 3 h was significantly higher in the DSS-vehicle group (n = 11) compared to the Control-clodronate (n = 14, p = 0.016) and DSS-clodronate groups (n = 12, p = 0.032). There was no significant difference between the Control-clodronate and DSS-clodronate groups (p = 0.363) and between the Control-vehicle and the Control-clodronate groups (p = 0.74) (**A**). Regarding wet fecal pellet weight, the same tendencies occurred, with significant differences between the DSS-vehicle and the Control-clodronate groups (p < 0.001) and the DSS-vehicle and DSS-clodronate groups (p = 0.007). Wet fecal pellet output of DSS-clodronate-treated mice was similar to Control-clodronate mice, with no significant difference (p = 0.114), just like in the case of Control-vehicle and Control-clodronate groups (p = 0.829) (**B**). When applying Evans blue through oral gavage, DSS-vehicle-treated animals (n = 6) showed significantly decreased transit times compared to both Control-clodronate (n = 8, p = 0.001) and DSS-clodronate-treated littermates (n = 6, p = 0.004). There were no significant differences detected between neither the Control-clodronate and DSS-clodronate groups (p = 0.249), nor the Control-vehicle and Control clodronate groups (p = 0.212). Metric data are shown as mean and corresponding standard deviation (SD). Statistical significance *p < 0.05; **p < 0.01, ***p < 0.001.

To confirm the role of infiltrating MMs in inducing dysmotility, we performed motility measurements in experimental mice. The weight of fecal pellets were counted in a fixed time frame as previously described^31^, but with an extended observation interval (3 h). Fecal pellets were obtained every 30 min and had their weight measured before and after drying in an oven for 6 h. Dry fecal pellet measurements reflect the raw material content of feces, including clotted blood, bacterial load and unprocessed food, whereas the amount of wet feces inform about the level of watery diarrhea associated with the colitis. For both measurements, fecal pellet weight returned to baseline level in the case of concurrent l-clodronate administration, with DSS-clodronate-treated animals producing a significantly decreased amount of feces compared to DSS-vehicle-treated mice (Fig. 4A,B). The difference was even more prominent in the case of wet feces (Fig. 4B). When measuring whole-gut transit time with Evans-blue albumin, DSS-vehicle-treated mice showed a significantly faster passage than controls, as expected, and the concurrent administration of l-clodronate prevented acceleration of GI motility in response to DSS treatment (Fig. 4C). In addition, l-clodronate treatment showed no significant affect in control mice, neither in fecal pellet weight, nor when performing Evans blue passage assays.

### DSS-colitis causes neuronal injury in the ENS alleviated by concurrent l-clodronate treatment

**Figure 5:**
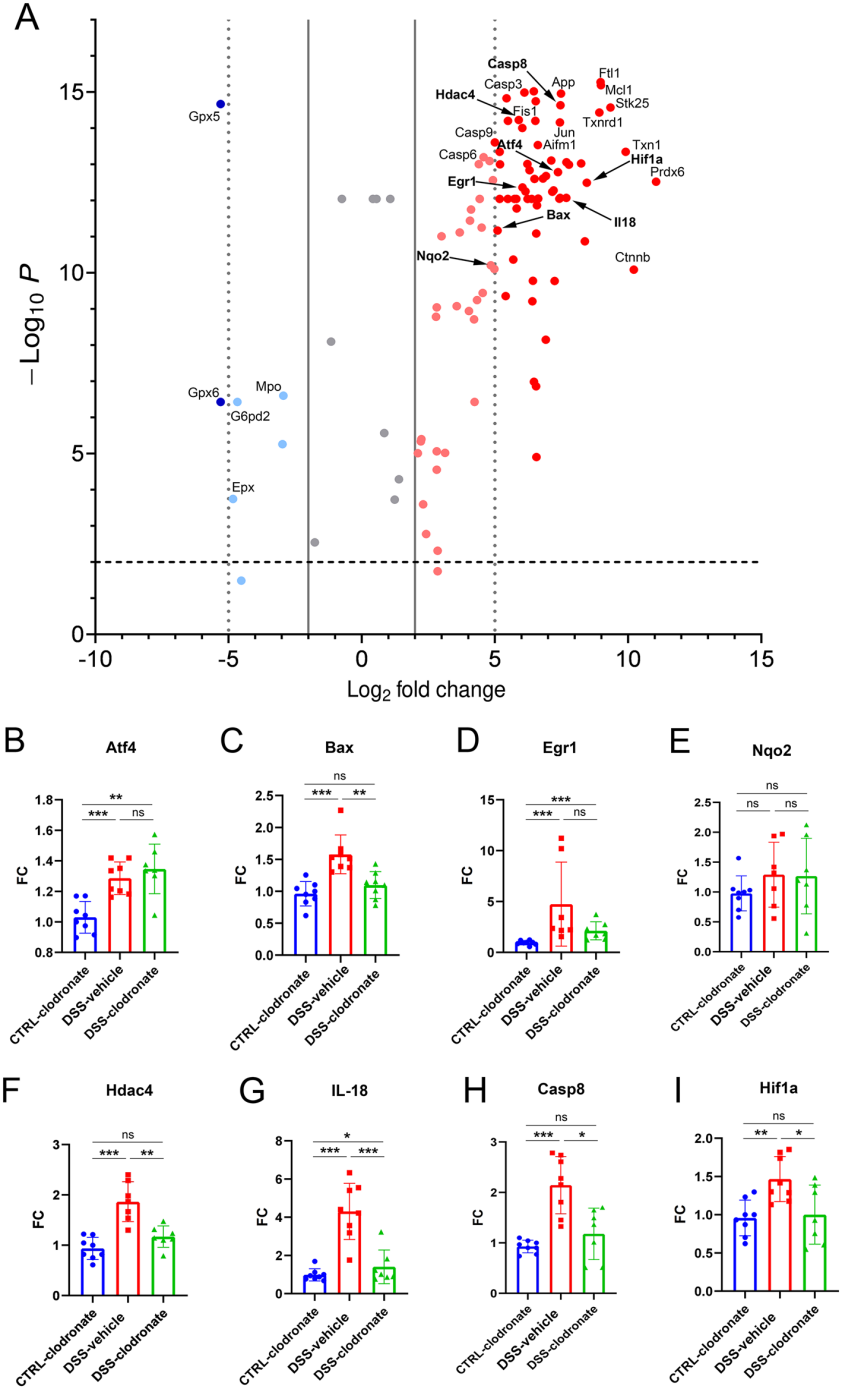
Expression of factors implicated in neuronal injury. Volcano plot shows DEGs selected from pathways of the GO biological processes database Response to hypoxia, Response to ROS, Neuron apoptotic process, Neuroinflammatory response and Neuron death (n = 107) after affinity propagation to remove overlapping of genes (**A**). Each dot represents a gene expressed in whole colon tissues of control (healthy) and DSS-treated experimental animals (n = 8); axis Y displays the log2-transformed adjusted p-values (Bonferroni correction). Axis X shows log2-transformed fold change (FC); positive FC value (red) reflects increased expression in DSS-treated, and negative FC value (blue) reflects increased expression in control mice. Genes with Log2 FC value > [2] are colored light red/blue, with > 5 are colored dark red/blue. Among DEGs annotated, bold displays genes specifically associated with neuroinflammation/degeneration (Atf4, Bax, Egr1, Nqo2, Hdac4, IL-18, Casp8) and Hif1 (**A**). For the latter genes, qPCR analyses were performed in isolated muscularis specimens of control- DSS-vehicle-treated and DSS-clodronate-treated animals (**B**–**I**). Expression of Atf4 (p < 0.001), Bax (p < 0.001), Egr1 (p < 0.001), Hdac4 (p < 0.001), IL-18 (p < 0.001), Casp8 (p < 0.001) and Hif1 (p = 0.002), but not of Nqo2 (p = 0.231) were significantly increased in DSS-vehicle-treated mice compared to controls. Concurrent l-clodronate treatment in DSS-treated animals decreased the expression of Bax (p = 0.003), Hdac4 (p = 0.002), IL-18 (p < 0.001), Casp8 (p = 0.014) and of Hif1 (p = 0.024) significantly, but not of Atf4 (p = 0.42), Egr1 (p = 0.208) and of Nqo2 (p = 0.99). Metric data are shown as mean and corresponding standard deviation (SD). Statistical significance *p < 0.05; **p < 0.01, ***p < 0.001.

Bulk RNA-seq analysis was performed comparing whole gut samples from the colon of control- vs DSS-treated mice, where the same DSS-treatment protocol was applied, but without the administration of l-clodronate or control liposomes, as previously described. In total, we identified n = 2225 differentially expressing genes (DEGs) after pooling samples (n = 8/group) from the same experimental groups (SFig 3). Next, we selected DEGs associated with Response to hypoxia, Response to ROS, Neuron apoptotic process, Neuroinflammatory response and Neuron death according to the Gene Ontology (GO) biological processes pathway database to identify DEGs that might be associated with neuronal injury detected in UC and murine colitis^20,32^ Fig. 5A**,** labeled genes). Because many of these DEGs are not specific to neural cells, we further selected genes that are highly neuron-specific and have unequivocally been associated with neuronal damage or degeneration, supported by the literature, including Activating transcription factor 4 (Atf4), Bcl-2-like protein 4 (Bax), Early growth response protein 1 (Egr1), N-Quinone Reductase 2 (Nqo2), Histone-deacetylase 4 (Hdac4), IL-18, and Caspase-8 (Casp8) (Fig. 5A, bold labeled genes). To validate the increased expression of these genes, specifically in the muscularis, we isolated the colon muscularis of control- DSS-vehicle-treated and DSS-clodronate-treated mice and performed qPCR for the 7 selected genes and Hypoxia Inducible factor 1 (Hif1), to assess the role of hypoxia, as a key pathogenetic factor in intestinal inflammation and consequent enteric neural damage^24,33–35^.

Atf4 expression was significantly increased in the muscularis of DSS-vehicle-treated vs control animals, but l-clodronate treatment did not return Atf4 expression to baseline (Fig. 5B). In contrast, the RNA expression of apoptosis regulator Bax increased significantly in DSS-vehicle-treated animals, but showed no difference in DSS-clodronate-treated mice compared to controls (Fig. 5C). Transcription factor Egr1’s expression was significantly increased in colitis and showed no difference in the case of concurrent l-clodronate injection (Fig. 5D). We detected no significant differences regarding Nqo2 expression in any comparison, that suggests that the muscularis is not a significant source of Nqo2 RNA during colitis (Fig. 5E). The expression of Hdac4, IL-18 and Casp8 showed a trend similar to Bax’s, where colitis-associated overexpression was diminished in the case of concurrent l-clodronate administration (Fig. 5F–H). Furthermore, expression of Hif1 showed the same trend (Fig. 5I), namely, MM-depletion during colitis decreased the hypoxia-related inflammatory response in the muscularis. Furthermore, our results suggest that colitis-associated overexpression of Atf4 and Egr1 cannot be reversed by MM depletion and l-clodronate treatment.

**Figure 6:**
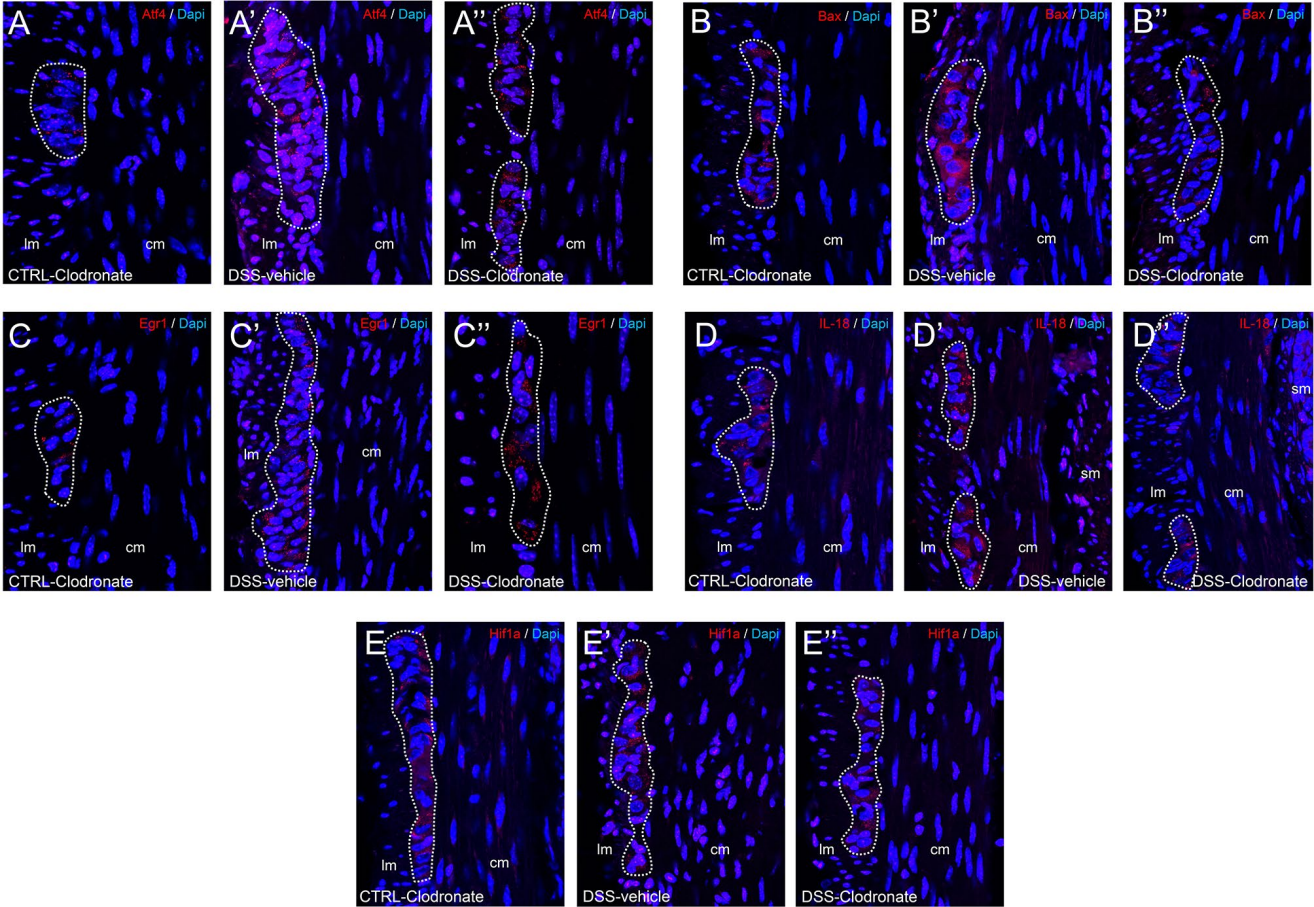
Tissue expression of Atf4, Bax1, Egr1, IL-18 and Hif1a proteins. Fluorescent immunostainings with Atf4 (**A**–**A**″), Bax1 (**B**–**B**″), Egr1 (**C**–**C**″), IL-18 (**D**–**D**″) and Hif1a (**E**–**E**″) antibodies show enteric ganglia indicated with dotted lines embedded between the longitudinal- and circular layer of the colon muscularis externa in different experimental setups. All proteins are present at a baseline level in enteric ganglia of control tissues (**A**–**E**) and show seemingly more intense staining in DSS-treated mice (**A**–**E**′). Enteric neural cells can be recognized by their large euchromatic nuclei visualized by 4′,6-diamidino-2-phenylindole (Dapi) staining. *Lm* longitudinal layer of muscularis externa, *cm* circular layer of muscularis externa.

To verify the protein expression of identified neurogenic factors in enteric neural cells we performed IHC with antibodies against Atf4 (Fig. 6A–A″), Bax1 (Fig. 6B–B″), Egr1 (Fig. 6C–C″), IL-18 (Fig. 6D–D″) and Hif1a (Fig. 6E–E″). We did not evaluate the in situ expression of Nqo2 and of factors Hdac4 and Casp8. Nqo2 showed no differential expression in any condition, and Hdac4 and Casp8 are constitutively expressed genes present in most cell populations. Immunostainings showed that enteric ganglia are the main source of Atf4, Bax1, Egr1, IL-18 and Hif1a proteins and staining intensity is increased in colitis (Fig. 6A–E″).

## Discussion

Our previous study has shown that the muscularis externa of the mouse colon undergoes significant morphological changes in terms of neuronal density, ECM patterning and immune cell infiltration during DSS-induced acute inflammation in mice. Furthermore, we found that the integrity of the impermeable myenteric plexus barrier was disrupted in the case of experimental colitis and fluorescent particles infiltrated the intraganglionic space^16^. This effect was reversible with the concurrent administration of l-clodronate and the depletion of MMs from the muscularis layer that indicate the crucial role of this cell population in the maintenance of barrier integrity in health and disease^16^. Neuronal or glial alteration and dysfunction in the ENS as a consequence of colitis were observed in multiple studies^36–38^, with the direct causes remaining unidentified. Therefore, we hypothesize that disruption of the myenteric plexus barrier might be responsible for long term dysmotility in IBD and other GI pathologies with acquired enteric neuronal dysfunction.

Previous studies reported achievement of macrophage-depletion by l-clodronate in the gut by inducing colitis, triggering inflammation-associated mononuclear recruitment^16,39^ or by depleting peritoneal macrophages^40,41^. By reducing the number of monocytes in the blood and bone marrow, l-clodronate prevents macrophage recruitment after the induction of DSS colitis, providing a suitable model to study colitis in the absence of macrophages^16^. We showed using flow cytometry that on the 5th day of the DSS-treatment (24 h after using l-clodronate) a marked drop occurred in the number of Ly6C+ monocytes. At this time the gut mucosa already showed signs of inflammation and increased macrophage infiltrates, but not the muscularis layer. Interestingly, l-clodronate did not effect granulocytes. Moreover, we did not detect a considerable decrease in Ly6C^low^ lymphocytes^42,43^, but l-clodronate treatment reduced the number of Ly6C^low^ and “negative” monocytes without a full depletion, that was observed in the case of Ly6C^high^ monocytes.

We recapitulated that l-clodronate-mediated depletion of MMs provides a suitable model to study muscularis- and enteric neuroinflammation in the absence of macrophages and that reduction of the colitis-associated mononuclear infiltration has functional consequences concerning gut motility. We showed that MM-depleted mice exhibited a colon transit time similar to control animals despite DSS-treatment and showed an intermediate colitic phenotype regarding colon length, disease activity and mucosal macrophage infiltration. Of note, despite the fact that DSS + l-clodronate treated mice showed unequivocal signs of inflammation (mainly in the mucosa and submucosa layers), the dysmotility-associated shortening of intestinal transit times were completely reversed to baseline level in the absence of MMs. This effect might be mediated by the disruption of the myenteric plexus barrier, that we described in our previous study^16^, where l-clodronate treatment decreased the degradation of circum-ganglionic ECM barriers by 60%^16^. Furthermore, based on the results of bulk-RNA sequencing of whole colon from control vs DSS-treated mice, we identified differentially expressed genes related to neuronal inflammation, injury or degeneration and validated their RNA-expression by qPCR using isolated muscularis samples of control- DSS-treated and DSS + l-clodronate treated animals.

Measurement of intestinal transit time is an essential physiological test to evaluate neuronal motor- and smooth muscle function in experimental animals. Although state-of-the-art ex vivo approaches based on video imaging (Gastrointestinal Motility Monitor, GIMM), or electrophysiology^44^ are already widely available to assess intestinal motility, in vivo methods using preclinical models can provide efficient and cost-effective alternatives^45^. Counting fecal pellets or detecting non-absorbable dyes are still frequently used non-invasive methods to assess whole gut transit times in various experimental settings^46–48^. Furthermore, hypoxic conditions, traumatic removal of gut segments (release of sympathetic mediators) and the severance from extrinsic innervation present in ex vivo models might alter normal physiology^45^. Moreover, to mitigate stress-induced motility alterations, we implemented a one-hour adaptation period for every mice after placing them in isolated cages before the initiation of motility measurements.

Our knowledge of mechanisms driving neuroinflammation is still developing, but studies on experimental colitis have provided insights into the disruptions caused by enteric neuroinflammation^4^. These disruptions include neuronal death, neurochemical plasticity, a reactive glial cell phenotype, neural hyperexcitability, local leukocyte infiltration to the enteric ganglia (plexitis), and disturbances in neurally regulated processes like intestinal motility^36,38^. Moreover, observations that plexitis in grossly uninflamed intestinal segments is a predictor of disease recurrence after surgery for Crohn’s disease underpin the role of muscularis inflammation in a clinical setting^48^. By isolating the muscularis layer of inflamed- control- and MM-depleted inflamed colon segments we could roughly evaluate the expression of neuron-derived mediators that derive from the ENS and are responsible for intestinal motility, eliminating mucosal contamination.

Bax1 is known for its involvement in mitochondrial outer membrane permeabilization, a key step in the intrinsic pathway of apoptosis. It promotes the release of cytochrome C from mitochondria, triggering a cascade of events leading to cell death^49^. It also was identified as one of the most prominent factors involved in neurodegeneration^50,51^, whereas Atf4 was reported as an unequivocally prodeath neuronal transcriptional factor, strongly implicated in Parkinson’s disease^52,53^, similarly to Egr1^54,55^. ROS-activation driven overexpression of Nqo2 has been identified in preclinical model and neuropsychiatric disorders^56,57^. Hdac4 was reported to play a pivotal role in the pathogenesis of ischemic stroke and post-stroke recovery by affecting neuronal death, angiogenesis, and neurogenesis^58^. Furthermore, Hdac4 was described to prevent white matter injury by modulating microglia/macrophage polarization^59^, an intriguing finding, given the fact that intraganglionic macrophages actively interact with enteric neural cells during colitis^5,12,16^. The emerging role of IL-18 in CNS pathologies, as ischaemic stroke and inflammation-driven neurodegeneration has been supported by multiple studies^60–62^ and Casp8 was reported in association with neuron-specific apoptotic processes and as a major pathogenetic factor in Alzheimer’s disease^63–65^. Researchers have highlighted the role of Hif1 in both cerebral ischaemia and in inflammation-associated brain damage^66,67^. From these factors, RNA expression of Bax, Hdac4, IL-18, Casp8 and Hif1 returned to baseline level compared to the colitis- associated upregulation, when MMs were depleted with l-clodronate during DSS-treatment.

To date, no studies reported Bax in association with the ENS, but Histone-deacetylases were shown to play a role in Hirschsprung-disease pathogenesis, though defective oxidative phosphorylation and impaired neurogenesis^68^. IL-18, however, is strongly implicated as a key factor in colitis^69^. Jarret and colleagues reported that neuron-derived IL-18 signaling has profound consequences on the mucosal barrier and invasive bacterial killing, and it controls tissue-wide intestinal immunity^70^ making IL-18 a crucial ENS-released mediator during inflammatory conditions. While Casp8 was not mentioned in connection with the ENS, the apoptosis-regulator was shown to contribute in the maintenance of the gut barrier by permitting inflammatory shedding and preventing necroptosis in response to mucosal pathogens^71^. Due to the fact that hypoxia was identified as an essential pathogenetic factor in intestinal inflammation and IBD^24,33–35^, we chose to assess Hif1 expression to reveal if MM-depletion and the presence of undamaged myenteric plexus barriers alleviates the hypoxia-induced stress reaction in the muscularis confirming our hypothesis. The protein expression of Atf4, Bax1, Egr1, IL-18 and Hif1a were also confirmed in enteric neurons at a tissue level using IF, where increased staining intensity was detected in DSS-treated mice.

Limitations of this study include the practical, but simplistic methodological approach to measure intestinal transit times: fecal pellet collection and Evans-blue assays only inform us about the motility of the whole intestinal tract, and we did not measure colon-motility separately. Of note, DSS-colitis only affects the distal segment of the large intestine and causes no inflammatory alterations in the coecum or small intestines^72^. Furthermore, bulk- or single-cell RNA-seq data specifically for the muscularis or for enteric neural cells were not available, and immunostainings for neurogenic factors in enteric ganglia were not double stained for neural markers, making the precise assessment of neural or glial origin difficult. Moreover, we have not assessed specifically the electrophysiological properties of barrier-degraded, or barrier-intact enteric ganglia, thus, we encourage further studies to comprehensively evaluate colitis-associated neuroinflammation and its functional consequences.

We conclude that MM-infiltration in the muscularis contributes to colitis-associated dysmotility and enteric neuronal dysfunction. l-clodronate-driven depletion of MMs is a suitable model to study inflammation in the muscularis devoid of macrophages. Moreover, DSS-treated mice administered concurrently with l-clodronate show milder clinical symptoms of colitis, intestinal transit times similar to control animals, and decreased expression of factors implicated in neural inflammation and death, including Bax, Hdac4, IL-18, Casp8 and Hif1a.

## Methods

### Animals

Male FVB/Ant mice (80–120 days old) were sourced from the Medical Gene Technology Unit, Institute of Experimental Medicine, Budapest. Mice were kept in a Specific Pathogen Free (SPF) environment at the Minimal Disease (MD) level, with 3–5 mice per cage. The housing conditions were carefully controlled, maintaining a temperature of 21 °C ± 1 °C, humidity at 65%, and a 12-h light–dark cycle. Mice were provided with unrestricted access to food and water. All experimental procedures strictly adhered to the guidelines established by the European Communities Council (86/609/EEC/2 and 2010/63 Directives of European Community). The protocol was approved by the Institutional Animal Care and Use Committee of the Institute of Experimental Medicine, Budapest, under permit number PEI/001/29-4/2013. This study has been conducted and reported in accordance with the ARRIVE (Animal Research: Reporting of In Vivo Experiments) guidelines, including euthanasia procedures where carbon dioxide (CO_2_) was employed as the chemical agent for euthanasia.

### Murine DSS-colitis model and l-clodronate treatments

Male FVB/Ant mice (100–120 days old) were treated with drinking water containing 3% Dextran sulphate sodium (DSS; MP Biomedicals, #160110) for 7 days. During treatment, DAI (Disease Activity Index) and weight were monitored. At the end of the experiment, animals were sacrificed by CO_2_ narcosis and cervical dislocation followed by colon length measurements. Distal colon samples were fixed in 4% PFA for histopathological assessment and immunostainings.

For clodronate treatments, DSS-treated mice and simultaneously, control animals were injected via tail vein with 200 μl of liposomal clodronate suspension (Clodronate Liposomes dissolved in PBS; LMS Consult GmbH & Co. KG) on the fourth day of the protocol to deplete infiltrating macrophages. Animals were relocated to the animal facility, where DSS administration continued for the DSS-clodronate-treated group for 4 additional days. Animals in the DSS-vehicle experimental group were injected with 200 μl of control liposomes as vehicle (Control (empty) Liposomes dissolved in PBS; LMS Consult GmbH & Co. KG). Experimental design of the study is shown in Fig. 1A.

### Flow cytometry

150 μl of mouse blood were stained with anti mouse CD45-PE-CY5.5 (Biolegend, #103131) and with anti Ly6c (Thermo Fischer, #25-5932-82) antibodies diluted 1:500, then incubated in 4 °C for 20 min and lysed with 2 ml BD FACS lysing solution. Cells were centrifuged on 400*g*, 6 min, then the supernatant was removed. Cells were rinsed with 2 ml PBS solution following by another centrifugation 400*g* 6 min. After removing the supernatant the cells were analysed in an additional 500 μl of PBS. The threshold was set on the SSC-FSC plot. Mononuclear cells (PBMCs) were gated in an SSC-CD45 plot. After selection of mononuclear cells monocytes were selected by either SSC-Ly6C plot and SSC-CD45 plot.

### Motility measurements

Experimental mice were placed individually in new bedding-free cages and monitored for 3 h after. Measurements for all mice started after one hour with no ad libitum food or water source as baseline point. Fecal pellets were collected and weighed every 30 min. Feces were dried at 50 °C for 6 h and re-weighed to collect dry fecal pellet weight data.

Assessment of whole gut transit time was carried out as previously described^31^. Briefly, mice were gavaged with 200 μl of the non-absorbable Evans Blue dye (Sigma-Aldrich). Period from the time of the gavage to the appearance of the first blue-colored fecal pellet was measured for every mice. Tests were ended at 600 min, mice that produced no Evans blue dyed feces until this time point were given 600 min as measurement value.

### RNA-seq library preparation and sequencing

RNA extraction from whole colon samples were prepared with the RNeasy Mini Kit (Qiagen) RNA Extraction and Isolation Kit. RNA integrity numbers (RINs) were determined with the BioAnalyzer Total RNA Nano6000 kit (Agilent, Santa Clara, CA, USA). Total RNA from the distal colon samples were converted into RNA-Seq libraries with the TrueSeq Stranded mRNA HT Sample Preparation kit (Illumina, San Diego, CA, USA). Sequencing was performed on an Illumina NextSeq500 instrument using the NextSeq500/550 High Output Kit v2.5.

### Quantitative real-time PCR

For qPCR analysis, the muscularis externa layer of the distal colon were separated mechanically from mucosal layers and were washed in DEPC-PBS and stored in − 80 °C freezer. Frozen tissue samples were homogenized in TRI reagent solution (Ambion), and total RNA was extracted using a QIAGEN RNeasy minikit (QIAGEN), as directed by the manufacturer. DNase I treatment was employed to remove genomic DNA contamination, and 100 ml of RNase-free DNase I (1 U of DNase) solution (Thermo Scientific) was added. NanoDrop 2000 was used to perform sample quality control and quantitative analysis. (Thermo Scientific). There was no evidence of amplification in the RT-minus controls. A high-capacity cDNA reverse transcription kit was used to create the cDNA. (Applied Biosystems). The Primer Ex-press 3.0 tool and Primer-BLAST software were used to create primers for the comparative Ct assays. Supplemental Table 1 shows the primers used in the real-time PCR reaction with Fast EvaGreen qPCR master mix (Biotium) on an ABI Ste-pOnePlus instrument (Applied Biosystems). The ABI StepOne 2.3 software was used to evaluate gene expression. Melt curve analysis on an ABI StepOnePlus PCR equipment was used to evaluate the amplicon. GADPH expression was used to standardize the results of the experiments.

### Histological procedures

Colon samples from experimental mice were fixed in 4% paraformaldehyde in PBS (PFA) for 24 h for immunofluorescence studies. The distal colon samples were obtained by removing the last 2 cm from the abdominal part of the large intestine in each mouse. Subsequently, the samples were thoroughly washed in PBS and then immersed in a medium containing 7.5% gelatin and 15% sucrose at 37 °C for 2 h. The tissues were rapidly frozen at − 60 °C using isopentane (Sigma). To prepare frozen sections for laser scanning confocal microscopy, the sections were cut at a thickness of 10 μm and collected on poly-l-lysine-coated slides (Sigma).

### Immunofluorescence and image analysis

For immunofluorescence staining, primary antibodies were diluted in 1% PBS-BSA. Frozen sections were incubated with primary antibodies for Agrin (R&D Systems, #AF550), Hu (abcam, #96474), F4/80(BM8) (Invitrogen, #41-4801-82), Col4 (abcam, #236640), Atf4 (Invitrogen, #MA5-32364), Bax1 (SCBT, #sc-7480), Egr1 (Invitrogen, #MA5-15008), IL-18 (Invitrogen, #MA5-47203) and Hif1a (Invitrogen, #PA116601) overnight at 4 °C, followed by secondary antibodies (Alexa Fluor 647 conjugated anti-rabbit IgG; Alexa Fluor 555 conjugated anti-goat IgG; Alexa fluor 488 conjugated anti-rat; Invitrogen) for 1 h. Cell nuclei were visualized by DAPI. Sections were covered with aqueous Poly/Mount (Polyscience Inc.) and examined with a Zeiss LSM 780 laser-scanning confocal microscope (Zeiss). Images were compiled by ImageJ and Adobe Photoshop CS6 software package.

### Cell counting and morphometry

Cell counting and morphometry analyses were conducted on images of sections obtained from distal segments of mouse colons using a Zeiss LSM 780 laser-scanning confocal microscope. To determine cell counts, tile scans of 9 images (3 horizontal, 3 vertical) at 20 × magnification with a resolution of 2 megapixels (2MP) were compiled from 2 spatially separated 10-μm-thick sections for every mouse using the ZEN software package. Results from individual sections of one specimen were averaged. This approach encompassed the entire cross-sectional area of the observed gut sample, as previously described in Dora et al.^16^. Morphometric analysis for cell density measurements was performed using the ZEN software package, with manual annotation of the measured areas. For cell counting of F4/80+ macrophages in the mucosa and muscularis layers, a systematic quantitative method was employed. This method involved software-assisted, manual cell counting performed by two independent observers, aided by the “cell counter” plugin of the ImageJ software package. The obtained cell counts were then compiled. To facilitate statistical analyses of cell density parameters, square micrometers (μm^2^) were converted to square millimeters (mm^2^).

### Statistical analyses

To assess normality we used the Shapiro–Wilk test and Bartlett’s test was used for testing homogeneity of variances. When comparing 3 groups, we used the Kruskal–Wallis test followed by uncorrected Dunn’s multiple comparison test. p-values < 0.05 indicate the significance and all p-values were two-sided. Differential gene expression panels were filtered for genes included in GO biological processes Response to hypoxia, Response to ROS, Neuron apoptotic process, Neuroinflammatory response and Neuron death and compiled with ggrepel (0.8.2), whereas volcano plot visualization was generated with EnhancedVolcano (1.8.0) R packages. Data were analyzed and graphs were generated with GraphPad Prism 9.1.1 for Windows, GraphPad Software, San Diego, CA.

### Animal studies

All experimental procedures strictly adhered to the guidelines established by the European Communities Council (86/609/EEC/2 and 2010/63 Directives of European Community). The protocol was approved by the Institutional Animal Care and Use Committee of the Institute of Experimental Medicine, Budapest, under permit number PEI/001/29-4/2013.

### Supplementary Information

Supplementary Information.

## Data Availability

The authors confirm that the data supporting the findings of this study are available within the article [and/or] its supplementary materials. Raw reads from the RNA-seq data of this study are available from the corresponding author, [DD], upon reasonable request.
