# Supplementary material for: Depletion of muscularis macrophages ameliorates inflammation-driven dysmotility in murine colitis model
Source: Sci Rep. 2023 Dec 17;13:22451. doi: 10.1038/s41598-023-50059-7 (PMC10725888; doi:10.1038/s41598-023-50059-7)
Supplement: Supplementary file 1 — Supplementary Information. [file 41598_2023_50059_MOESM1_ESM.docx]

**SUPPLEMENTARY MATERIAL**


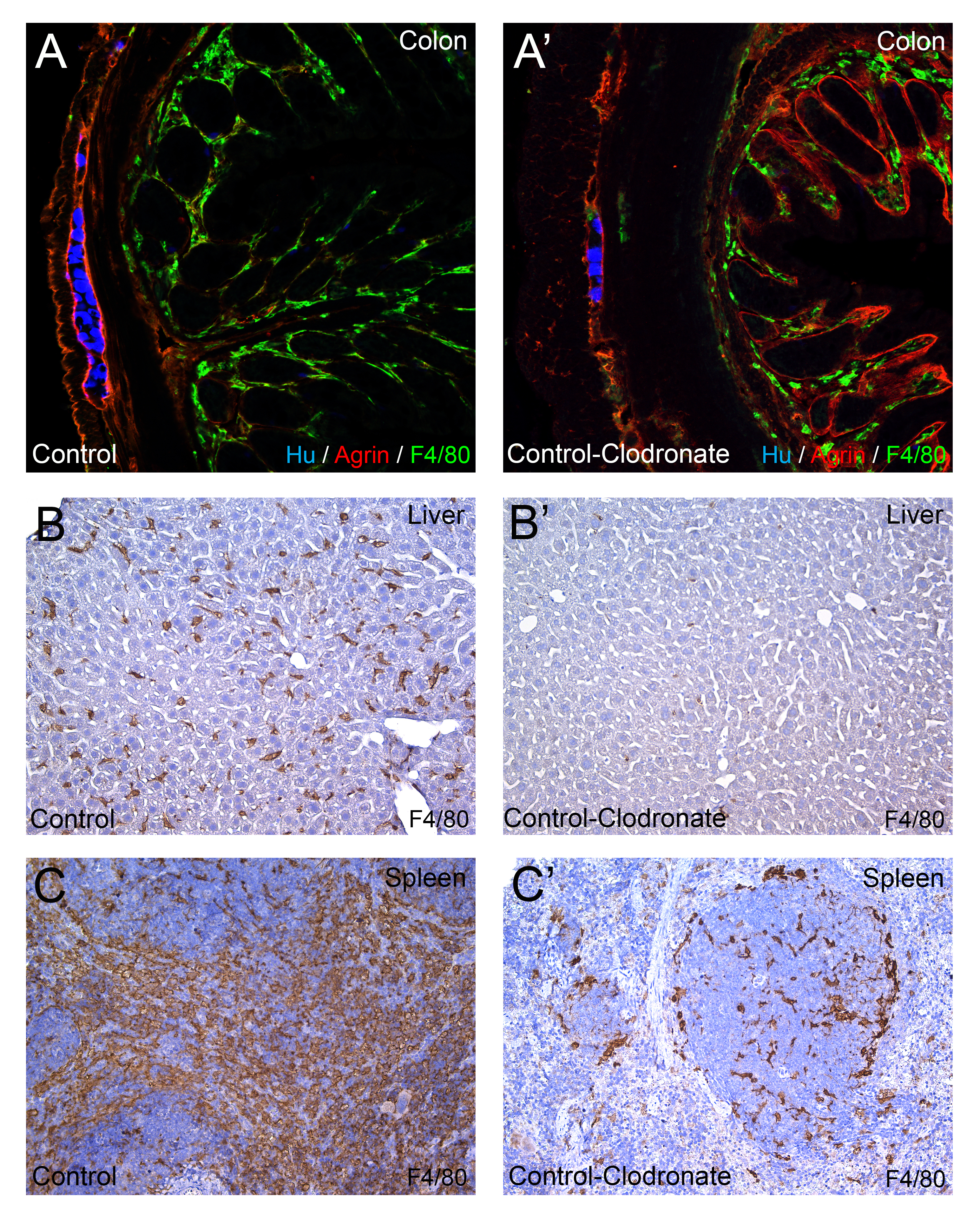


**Supplementary Figure 1. Comparison of Control and Control-clodronate-treated organs.** Immunofluorescence staining shows that no significant difference is present between the Control- and the Control-clodronate colon, concerning general morphology, integrity of the agrin-expressing myenteric plexus barrier, and F4/80+ macrophage infiltration in the mucosa or the muscularis (**A-A’**). Immunohistochemistry on liver and spleen tissues from the same animals demonstrate marked difference in the number of F4/80+ macrophages between Control- and Control-clodronate tissues, where the latter is present with notable reduction in the number of macrophages (**B-C’**).


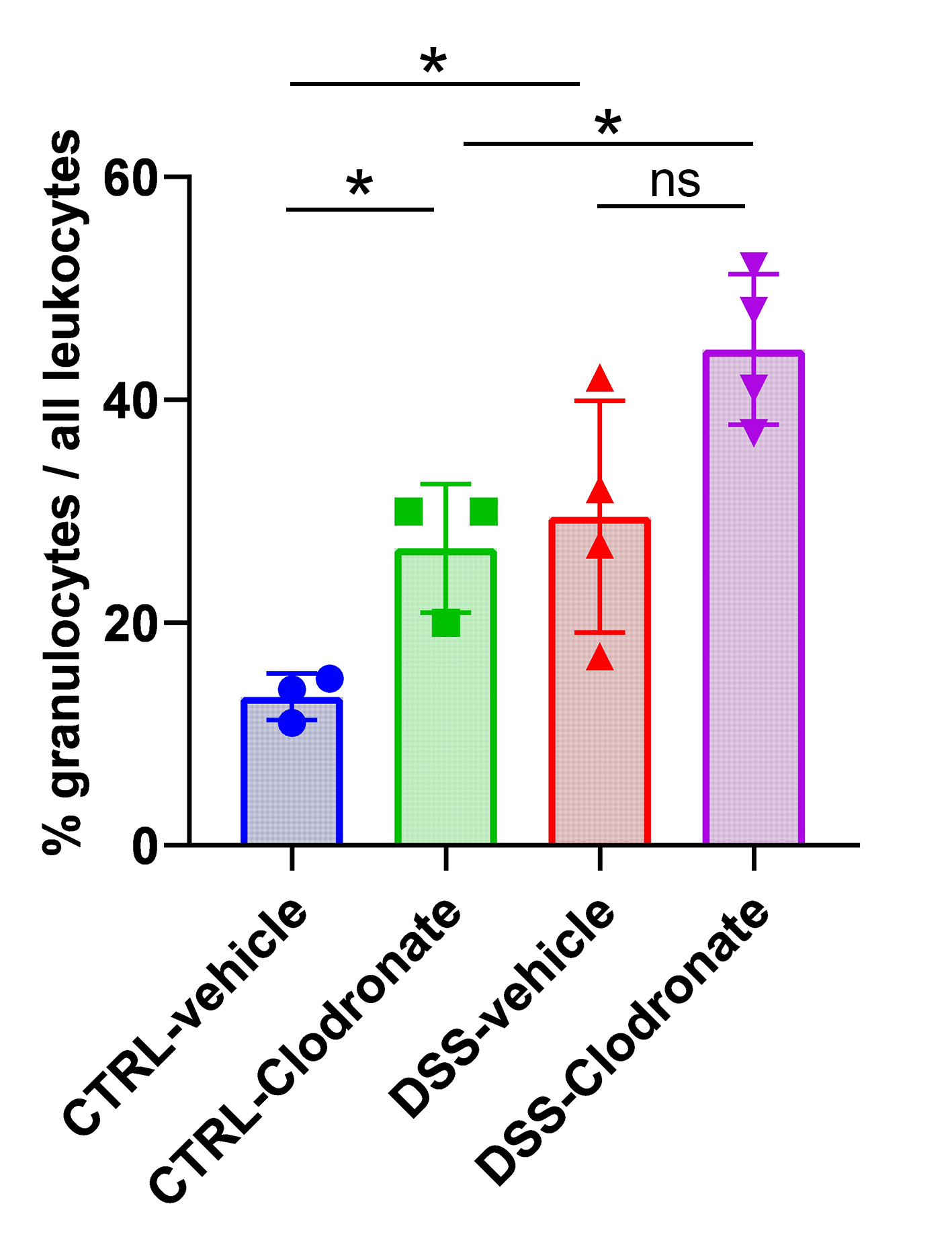


**Supplementary Figure 2. Quantification of blood granulocytes in the context of L-Clodronate treatment.** The percentage of granulocytes from all leukocytes was significantly higher in the Control-Clodronate group compared to the Control-vehicle group (p=0.044) and there was a trend when comparing the DSS-Clodronate group with the DSS-vehicle group towards the former (p=0.058). Granulocytes in the blood of DSS-vehicle-treated mice represented significantly higher percentage than in the blood of Control-vehicle-treated mice (p=0.049). There was a significant difference between the two L-Clodronate-treated group (CTRL vs DSS, p=0.014)


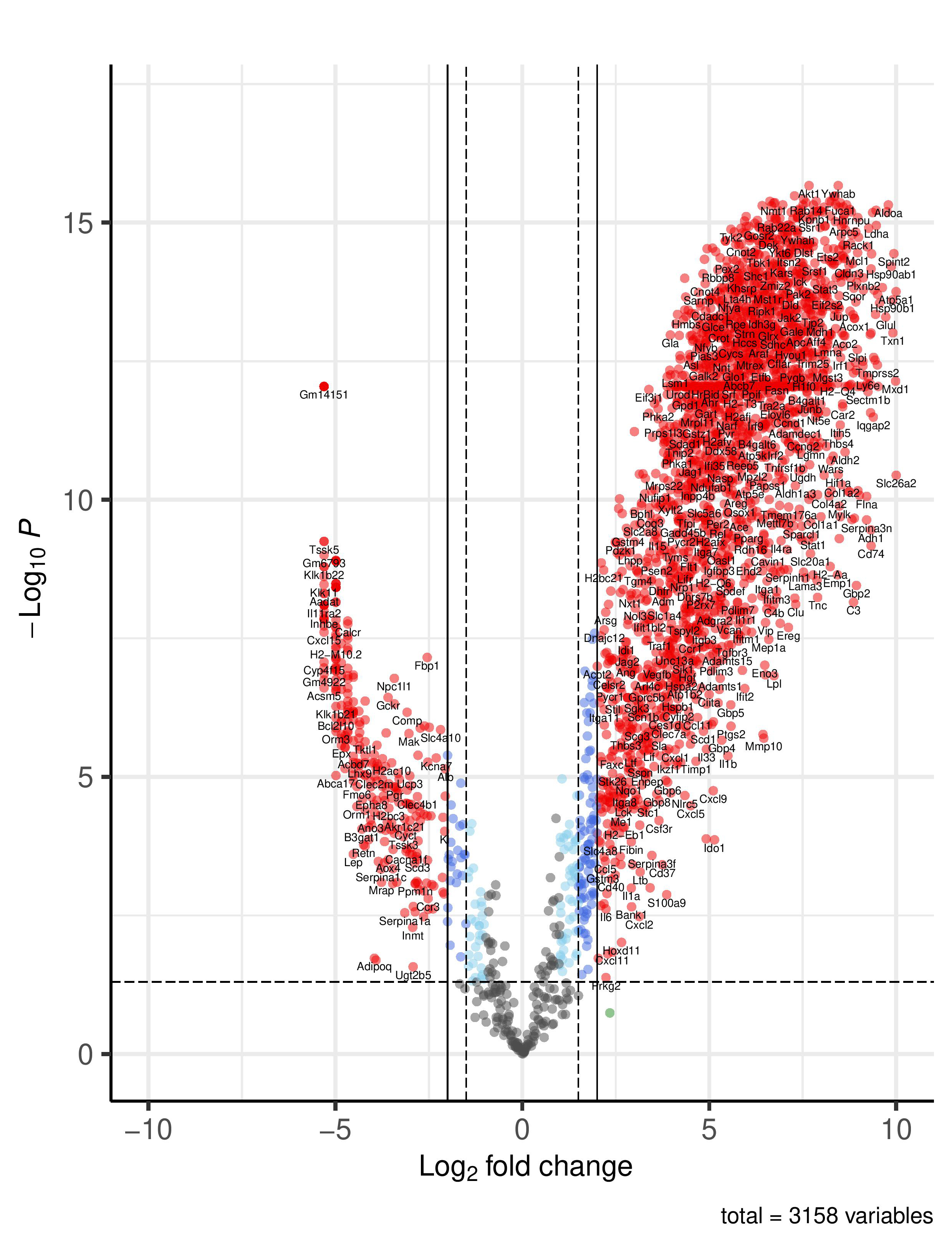


**Supplementary Figure 3.** Volcano plot of all detected genes (n=3158) according to bulk RNA-sequencing in DSS-treated vs. control mice colon. Each dot represents a gene; axis Y displays the log2-transformed adjusted p-values (Bonferroni correction). Axis X shows log2-transformed fold change (FC); Negative FC value reflects overexpression in control colon, positive FC value reflects increased expression in DSS-treated colon. Genes with Log2 FC value > [2] and a significant adjusted p-value (<0.05) are colored in red. Genes with significant adjusted p-value and Log2 FC value greater than [1.5], but lesser than [2] are colored in dark blue. Genes with significant adjusted p-value, but Log2 FC value < [1.5] are colored in light blue. Genes showing no significant difference between the two groups are colored in grey.

**Supplementary Table 1. Primer sequences used in qRT PCR reactions.**

gapdh f: TGACGTGCCGCCTGGAGAAA, r: AGTGTAGCCCAAGATGCCCTTCAG

atf4 f: GAATGGCCGGCTATGGATGA, r: TCTGTCCCGGAAAAGGCATC

nqo2 f: TATCACTGGTGCCCCCTCTA, r: GCACCCTATCCATCCAACCTT

casp8 f: CTCTCAGAAGAAGTGAGCGAGT, r: TGCCAGCATGGTCCTCTTCT

egr1 f: GCACCTGACCACAGAGTCCTTT, r: CGAGTCGTTTGGCTGGGATAA

hdac4 f: GCACAAATCCTCTCAACAGACA, r: GTCGCTATCCACCCCAACAC

bax f: GAACCATCATGGGCTGGACA, r: GGAGAGGAGGCCTTCCCAG

hif1a f: CGTGCTTGGTGCTGATTTGT, r: GAGCGGCCCAAAAGTTCTTC
